# Physical activity recommendations for health: what should Europe do?

**DOI:** 10.1186/1471-2458-10-10

**Published:** 2010-01-11

**Authors:** Pekka Oja, Fiona C Bull, Mikael Fogelholm, Brian W Martin

**Affiliations:** 1 UKK Institute, P.O.Box 30, 33501 Tampere, Finland; 2 School of Sport, Exercise and Health Science, Loughborough University and School of Population Health, The University of Western Australia, Stirling Highway, Nedlands, Western Australia, 6009, Australia; 3 Health Research Unit, Academy of Finland, Vilhovuorenkatu 6, POB 99, 00501 Helsinki, Finland; 4 Institute of Social and Preventive Medicine, University of Zurich, Hirschengraben 84, 8001 Zurich, Switzerland

## Abstract

**Background:**

Accumulating scientific evidence shows physical activity to have profound health benefits amenable to substantial public health gains. Accordingly, recommendations on how much and what kind of physical activity enhances health have been issued. The 1995 recommendation from the U.S. Centres for Disease Control and Prevention and the American College of Sports Medicine has been adapted worldwide, including Europe. Recently an extensive review of new evidence was undertaken and refined recommendations were issued by the U.S. Department of Health and Human Services. We summarise the development of physical activity recommendations and consider the need and possible ways to update the current European situation.

**Discussion:**

The new recommendations include several new elements when compared to the 1995 recommendation, the most notable being the greater emphasis on the contribution of vigorous-intensity activities, and the inclusion of activities for muscle strength and bone health. They also include specific recommendations for young people, middle-aged adults, older adults and some special groups. The existing Pan-European and national physical activity recommendations in Europe are mostly based on the 1995 recommendation and primarily target adults and young people. Thus the degree to which they are compatible with the new recommendations varies. In view of the growing public health importance of physical activity, we discuss the need to review the existing physical activity recommendations at the European level and assess their consistency with the new evidence and the new recommendations.

**Summary:**

We argue that a review of the current physical activity recommendations in Europe should be undertaken in view of the most recent research evidence. We recommend that such a task should be taken on by WHO Europe in parallel with the ongoing work by WHO global Headquarters. Following this, each country should develop communication strategies and implementation guidelines that take into account their ethnic and cultural diversity.

## Background

As the scientific evidence of the health benefits of physical activity has become more established concentrated efforts have been made to inform the policy makers, relevant professionals and the general public on how to implement this knowledge for improved health. These messages have taken two general forms. Guidelines are typically population-targeted consensus statements on the general course of actions and strategies that inform how best implement successful interventions to realise the health potential of physical activity. Recommendations, on the other hand, are summary statements, based on the latest evidence, communicating the necessary dose of physical activity required for specific and general health gains. Recommendations can be specific to a population group (adults, older adults, young people) and can be either aimed at primary prevention or secondary prevention (treatment of those with existing conditions).

The most well known evidence-based physical activity recommendations for public health was issued more than a decade ago by the U.S. Centres for Disease Control and Prevention (CDC) and the American College of Sports Medicine (ACSM) [1]. It read: "Every U.S. adult should accumulate 30 minutes or more of moderate-intensity physical activity on most, preferably all, days of the week". This recommendation has been widely adopted in Europe and worldwide. Recently two extensively documented evidence-based physical activity recommendations have been issued in the United States: the first by the American Heart Association (AHA) & the American College of Sports Medicine [2,3] and the second by the U.S. Department of Health and Human Services (U.S. DHHS) [4]. In this short communication we briefly outline the development of physical activity recommendations, summarise the latest recommendations and consider the need and possible ways to incorporate the new information effectively in pan-European and national health policies in Europe.

## Discussion

The World Health Organisation (WHO), the European Union and several European countries have recently begun to consider physical activity in their health policies. WHO in its Global Strategy on Diet and Physical Activity [5] called for member states to include physical activity in their national health policies. This strategy was followed by a guide for population-based approaches to increasing levels of physical activity [6]. Also the WHO Regional Office for Europe has addressed the physical activity issue in a number of recent documents including a European framework to promote physical activity [7] and a call for counteracting obesity [8]. The most specific overview at the European level exists in the form of the WHO publication "Physical activity and health. Evidence for action" [9]. This documents states that there are currently no officially recommended levels of physical activity for the European region, but it cites the international recommendations, it gives an overview of the health effects and public health relevance of physical activity and it explains the principles of physical activity promotion.

Also the European Union has taken physical activity into their health policies. In 2000, a project financed by the European Commission described and analysed the then existing national approaches in physical activity promotion in Europe [10]. In 2007, the White Paper on a Strategy for Europe on Nutrition, Overweight and Obesity [11] called the Member States and the Union to take pro-active steps to stop the decline in physical activity levels. As one concrete outcome the EU Platform for Action on Diet, Physical Activity and Health was established [12]. This forum includes representatives from European scientific, industrial and civic organisations. More recently the Sport Unit of the European Union's Directorate for Education and Culture in its White Paper on Sport [13] considered health as the key social justification for sports, and is currently in a process to issue policy recommendations for physical activity promotion in the member countries [14].

The European countries have responded to these calls for policy development in a variety of ways. A recent study [15] identified 49 national policy documents on physical activity promotion from 24 European countries. Twenty-two national and 5 sub-national documents from 14 countries were published in English and were included in a content analysis. Out of the 27 documents, "only 6 policies contained quantified physical activity goals specifying the intended level of physical activity to be achieved over a specific time period". Where recommended physical activity doses are used, they follow in general the 1995 CDC & ACSM recommendation of 30 minutes of moderate-intensity physical activity, although there is much diversity how this message is presented. For instance, the Finnish recommendation is illustrated by a physical activity pie [16], and the Swiss recommendation by a pyramid [17], while the UK recommendation is presented in a written form [18]. Most of the European countries had recommendations for children and adolescents and for adults and only a few for elderly people and for other special groups.

Evidence-based physical activity recommendations provide guidance for policy development and physical activity promotion. In more than a decade after the 1995 CDC & ACSM physical activity recommendation [1] much new scientific evidence on physical activity and health has accumulated. The United States has again led the way in undertaking a major systematic review of evidence and providing updated recommendations, first by the American Heart Association and the American College of Sports Medicine [2,3] and most recently by the U.S. Department of Health and Human Services [4]. Also, in Canada a process is underway to review the evidence and revise where necessary national guidelines [19] with a recent consensus conference held in 2009. These new developments highlight the need to consider the possible consequences for the relevant health policies in Europe.

According to the 2007 AHA & ACSM recommendation for adults "To promote and maintain health, all healthy adults aged 18-65 years need moderate-intensity aerobic physical activity for a minimum of 30 min on five days each week or vigorous-intensity aerobic activity for a minimum of 20 min on three days each week". Also, it notes specifically that combinations of moderate- and vigorous-intensity activity can be performed to meet this recommendation. In addition, these new recommendations include a clear statement on the contribution of activities that increase or maintain musculo-skeletal health and note that increased amounts of physical activity over and above the recommended amount might be required to prevent increases in body weight. The recommendation for older adults is similar to the adult recommendation with additional considerations for fitness level and special activities for flexibility and balance.

This new recommendation does not refute the 1995 recommendation, but it does include some new specifications as well as several new elements when compared to the 1995 recommendation. The most notable new elements are the greater emphasis on the contribution of vigorous-intensity activities, and the inclusion of activities for muscle strength and bone health. It can be said that while the 1995 recommendations invited life-style activities as health-enhancing physical activity the 2007 recommendation posits that more vigorous exercises and sportive activities are also health promoting.

The most recent physical activity recommendations were issued by the U.S. Department of Health and Human Services [4]. These "2008 Physical Activity Guidelines for Americans" are based on extensive systematic review and critical analysis of the current evidence. They include specific recommendations for young people, middle-aged adults and for older adults (see table 1), and also for people with disabilities and for pregnant and postpartum women.

**Table 1:** U.S. Department of Health and Human Services physical activity recommendations [4].

Target group	Recommendation
Children and adolescents (aged 6-17)	Children and adolescents should do 1 hour (60 minutes) or more of physical activity every day. Most of the 1 hour or more a day should be either moderate- or vigorous-intensity aerobic physical activity. As part of their daily physical activity, children and adolescents should do vigorous-intensity activity on at least 3 days per week. They also should do muscle-strengthening and bone-strengthening activity on at least 3 days per week.

Adults (aged 18-64)	Adults should do 2 hours and 30 minutes a week of moderate-intensity, or 1 hour and 15 minutes (75 minutes) a week of vigorous-intensity aerobic physical activity, or an equivalent combination of moderate- and vigorous-intensity aerobic physical activity. Aerobic activity should be performed in episodes of at least 10 minutes, preferably spread throughout the week. Additional health benefits are provided by increasing to 5 hours (300 minutes) a week of moderate-intensity aerobic physical activity, or 2 hours and 30 minutes a week of vigorous-intensity physical activity, or an equivalent combination of both. Additional health benefits are gained by engaging in physical activity beyond this amount Adults should also do muscle-strengthening activities that involve all major muscle groups performed on 2 or more days per week.

Older adults (aged 65 and older)	Older adults should follow the adult guidelines. If this is not possible due to limiting chronic conditions, older adults should be as physically active as their abilities allow. They should avoid inactivity. Older adults should do exercises that maintain or improve balance if they are at risk of falling.

Importantly, the U.S. DHHS recommendations target also children and adolescents. Accordingly, children and adolescents aged 6-17 years should do at least one hour physical activity every day, which should include moderate- and vigorous-intensity aerobic activities and muscle-strengthening and bone-strengthening activities. This recommendation of at least one hour of daily physical activity is compatible with several recent U.S. and an Australian recommendation but less than the Canadian recommendation of 90 minutes physical activity per day [20]. Many earlier recommendations for young people have been based on limited evidence [21]. Still health effects in adults are better documented than in children, but the U.S. DHHS recommendation is informed by new carefully assessed evidence.

The recommendations for adults and older adults are in principal similar to the 2007 recommendations, but the focus is on total weekly time (150 minutes) rather than a specification of one recommendation addressing the number of sessions par week (five times 30 minutes per week) This shift in presentation of the amount of activity required provides important flexibility for individuals to accumulate or spread their weekly activity in varied and convenient ways. However, whilst it emphasises that at least three weekly sessions helps to avoid too large single doses, which may cause overstrain, it does not preclude the interpretation that the weekly dose can be implemented in diverse ways best fitting one's individual needs.

As general principles the recommendations state:

- some activity is better than no activity

- many health benefits increase with the increase of the intensity, frequency and/or duration of activity

- the health benefits of physical activity greatly outweighs the health risks

- the health benefits of physical activity are largely independent of gender, race and ethnicity

It is important to note that various supporting publications were issued along with the U.S. DHHS recommendations, each tailored differently for specific target groups. For examples, in addition to the recommendations, there was a guideline for policy makers and release of the full scientific report from the Scientific Advisory Committee aimed at helping the health professionals and researcher community. Although the US guidelines includes a toolkit for "building awareness and participation" in communities, it does not explicitly cover "putting the guidelines into practice". Successful dissemination and implementation of physical activity guidelines is likely to be enhanced with provision of these kinds of supporting materials.

Is there a need for new or updated European physical activity recommendations? Currently the World Health Organisation is in the final stages of developing global physical activity recommendations. The thoroughness of the review process undertaken for the recent U.S. recommendations practically precludes any major discrepancies in the scientific conclusions, and since the used evidence is global in nature, there is no reason to believe that it should not be applicable for European populations as well. So the question is, what do these updated evidence-based physical activity recommendations mean from the European perspective?

There are many international players in the field of physical activity promotion for health in Europe. They include WHO Europe, the European Union, the European network for the promotion of health-enhancing physical activity (HEPA Europe) as well as several European public health and sport organisations. Several documents from these organisations have already recognised the public health relevance of physical activity, and some of them have addressed aspects of physical activity promotion. The soon to be issued physical activity guidelines by the Sport Unit of the directorate-general education and culture of the European Commission [14] will add another important perspective. These guidelines focus on European and national policy development rather than informing directly the public.

Europe consists of more than 60 nations of diverse ethnic, cultural, religious, political and socio-economic background. The health benefits of physical activity warrant that physical activity is included in national health policies across the continent, but only about one third of the countries has physical activity policies and only a small proportion of them seem to use recommended levels of physical activity for defining and evaluating goals at the policy level. Furthermore, many of the existing national physical activity recommendations are based on the 1995 CDC & ACSM recommendation, and they may need to be updated based on the newest evidence as reviewed for the U.S. DHHS 2008 physical activity recommendations.

In view of the growing public health importance of physical activity, it would be useful to review existing physical activity documents at the European level and to verify the existence and content of recommendations for levels of physical activity as well as of guidelines for the implementation of physical activity promotion. Given the deficiency of physical activity policies for public health among the European countries, it will be even more important to do the same at the national level. Cultural differences between and within European countries will - and probably should - lead to some differences in the presentation of national recommendations, but they should be compatible with the latest available evidence.

The potential audiences for any new or revised physical activity recommendations are many: politicians, policy makers, researchers, professionals and the public. In order to communicate effectively to these diverse target groups, the style, content and communication channel should be tailored according to their specific needs. Researchers, professionals and other experts should be provided with a comprehensive summary of the latest evidence. Policy and decision makers at different levels need clear and concise facts about the public health benefits of physical activity and guidelines on how to design effective strategies and policies. The non-governmental civic organisations should be given information which encourages them to include physical activity in their activities, and again guidelines on how they might accomplish this and contribute to public health efforts. Communication messages aimed at the general public need to be simple and clear, culturally appropriate and target group-specific to support people's personal behavioural choices.

## Summary

We believe that a review of the current national physical activity recommendations in each country within the European region should be undertaken in view of the most recent research evidence. We suggest that such a task should be led or coordinated by WHO Europe, and performed in parallel with WHO global headquarters ongoing work to avoid duplication and harmonize the process and outputs. This would allow the core components of activity dose (frequency, intensity, duration, mode) to be applicable for all countries in the region. Following this, each country should develop communication strategies to disseminate their recommendation and compliment these with guidelines on how to promote physical activity and implement evidence based interventions and policy. These implementation guidelines should take into account the ethnic and cultural diversity in each European country. Involving relevant stakeholders such as policy makers, health professionals, other sectors and civic organisations would help maximise adoption, implementation and dissemination of the recommendations.

## Competing interests

The authors declare that they have no competing interests.

## Authors' contributions

PO wrote the first draft text on which the other authors commented. All authors read and approved the final manuscript.

## Author's information

All authors have contributed to research on physical activity and health and have served as experts for the World Health Organisation, the European Union and their respective national health authorities in this field.

## Pre-publication history

The pre-publication history for this paper can be accessed here:

http://www.biomedcentral.com/1471-2458/10/10/prepub
